# Multi-institutional experience treating patients with cardiac devices on a 1.5 Tesla magnetic resonance-linear accelerator and workflow development for thoracic treatments

**DOI:** 10.1016/j.phro.2024.100680

**Published:** 2024-11-26

**Authors:** Rick Keesman, Erik van der Bijl, Linda G.W. Kerkmeijer, Neelam Tyagi, Osman Akdag, Jochem W.H. Wolthaus, Sandrine M.G. van de Pol, Juus L. Noteboom, Martijn P.W. Intven, Martin F. Fast, Astrid L.H.M.W. van Lier

**Affiliations:** a Department of Radiation Oncology, Radboud University Medical Center, Nijmegen, The Netherlands; b Department of Medical Physics, Memorial Sloan-Kettering Cancer Center, NY, USA; c Department of Radiotherapy, University Medical Center Utrecht, Utrecht, The Netherlands

**Keywords:** CIED, MRgRT, MR-linac

## Abstract

**Background and purpose:**

Patients with cardiac implantable electronic devices (CIED patients) are often ineligible for online magnetic resonance-guided radiotherapy (MRgRT), most likely due to the absence of established guidelines. Existing radiotherapy (RT) and magnetic resonance imaging (MRI) guidelines offer an opportunity to construct MRgRT protocols, promoting equitable access. Our objective was to present such a workflow, share multi-institutional experiences treating CIED patients with MRgRT on a 1.5 T magnetic resonance-linear accelerator (MR-linac), and investigate geometric accuracy and electrocardiogram (ECG) monitoring for thoracic treatment.

**Materials and methods:**

A risk analysis identified strategies for safe MRgRT for CIED patients. At three institutions, 21 pelvic and abdominal patients were treated. Patient records were analyzed for adverse events. Geometric accuracy was investigated using B_0_-mapping with a phantom simulating moving lung and cardiac lesions near a CIED. Volunteer measurements evaluated the effects of patient positioning and MRI sequences on ECG signal distortion.

**Results:**

MRI and RT workflows were adaptable to MRgRT. No adverse events were recorded. B_0_-maps showed a maximum mean difference between static and dynamic phantom configurations of 0.1 mm, increasing to 0.4 mm distortion in the presence of a CIED. ECG readings exhibited severe distortions during scanning, hampering heart rhythm detection for most MRI sequences.

**Conclusions:**

CIED patients can safely undergo treatment on a 1.5 T MR-linac following RT and MRI guidelines. For targets near CIEDs, a B_0_-mapping procedure was considered accurate enough to determine MRgRT eligibility. Pulse oximetry is recommended for cardiac monitoring during MRI scanning due to ECG signal distortion.

## Highlights

• Patients with a cardiac implantable electronic device can safely undergo MRgRT.
• B0-mapping can be used to determine treatment eligibility of thoracic patients.
• ECG might be heavily distorted during imaging making pulse oximetry indispensable.

## 1 Introduction

Since the introduction of magnetic resonance-linear accelerators (MR-linacs), online magnetic resonance-guided radiotherapy (MRgRT) has been used to treat an increasing number of disease sites [1]. Patients with a cardiac implantable electronic device (CIED) are typically excluded from MRgRT, most likely due to the absence of established guidelines or logistical challenges. Guidelines for both radiotherapy [2], [3] (RT) and magnetic resonance imaging [4], [5] (MRI) exist, however, to mitigate inherent risks (e.g., CIED malfunction and severe tissue heating). Cardiac monitoring, utilizing an electrocardiogram (ECG) and/or pulse oximeter, is still considered essential during MRI examinations, to ensure patient safety.

For patients with a moving target, two types of MRI sequences are particularly beneficial; motion monitoring (MM) sequences and acquisitions that are robust against motion artifacts [6], [7]. While applicable across various anatomical sites, these sequences are especially valuable when respiratory motion affects the target and surrounding tissues [8]. Gating, based on MM images, allows irradiation only during specific phases of the breathing cycle, thus reducing dose to organs at risk thereby potentially improving outcomes or reducing toxicity [9], [10].

Recently, pelvic and abdominal cancer patients, with disease sites distant from the CIED and leads, have been treated on a 1.5 T MR-linac [11]. Cardiac radioablation has been conducted using a 0.35 T MR-linac [12], [13], targeting sites near the CIED and leads but with limited expected interplay between MRI and CIED and leads because of the low magnetic field strength. Utilizing a 1.5 T MR-linac for patients with a CIED close to the treatment site (e.g., thorax) demands heightened scrutiny due to the potential impact of the CIED and leads on image quality. While signal voids can render MRI ineffective, unnoticed geometric distortions may result in image deformation and thus incorrect delineations, ultimately leading to mistreatment [14], [15]. While there are strategies to reduce these effects [16], [17], [18], they are not readily available on the 1.5 T MR-linac system, presenting a significant limitation.

An alternative approach involves mapping B_0_-inhomogeneities [19], which can be used to determine whether geometric distortions require further action (e.g., changing beam-setup, expanding margins, or opting for conventional treatment), and has been used for patients with passive implants [20], [21]. Motion during MRI acquisition can affect image quality [22], but whether such a B_0_-mapping procedures can be extended for thoracic patients, with moving targets and/or implants, remains, to the best of our knowledge, unexplored.

It is also unclear what the effects of MM or motion compensated acquisitions are on the ECG signal in an online MRgRT setting, potentially diminishing its diagnostic value [4], [23]. Consequently, the added benefit of ECG over pulse oximetry for MRgRT in thoracic patients with a CIED remains uncertain when using these sequences.

This manuscript has two primary objectives. Firstly, to report on a risk-analysis derived workflow of online MRgRT for patients with a CIED and share multi-institutional experiences treating such patients on a 1.5 T MR-linac. Secondly, to extend the workflow for patients with treatment sites close to their CIED. To that end, the applicability of B_0_-mapping procedures for thoracic patients and the level of ECG signal distortions during the acquisition of commonly available MRI sequences were investigated.

## 2 Materials and methods

### 2.1 Risk analysis

Three institutions (Radboudumc (NL), UMCU (NL), and MSKCC (USA)) performed their own clinical implementation, tailored to the local situation. In all cases, a failure mode and effects analysis (FMEA) was performed by a multidisciplinary team and cross-departmental collaboration was required. Guidelines for RT and MRI formed the starting point for treating CIED patients with a CIED with a 1.5 T MR-linac. Differences between online MRgRT for patients with a CIED, with respect to conventional RT and MRI workflows, were identified. All key differences, which required workflow adaptation for online MRgRT, were included.

The prospective FMEA resulted in six distinct differences between online MRgRT and MRI and RT as separate modalities.

1. Online MRgRT is a more resource intensive treatment for patients with a CIED than MRI and RT separately because of the required patient monitoring and CIED examination before and after each fraction.
2. Most MRI scanners have an option that changes sequences to automatically adhere to the set MRI conditions. On the Elekta Unity, however, such an option is not currently available.
3. Online MRgRT consists of a repetition of treatment fractions that are kept largely the same per treatment, in terms of dose, patient positioning, CIED, and sequences used.
4. For disease sites in the vicinity of the CIED or leads, geometric distortions of the MR images might lead to reduced treatment quality and, other than falling back on pre-treatment computed tomography information, there seemed to be no validated method of estimating the severity of distortions in this setting.
5. There is a greater distance to the patient, compared to a typical MRI environment, and no direct view from the console room during treatment.
6. The impact of various sequences and patient positioning on ECG signal distortion, and thereby the efficacy of ECG monitoring is unknown for the online MRgRT setting.

Two information gaps related to treatment near CIEDs were identified during the FMEA. First, we investigated whether a B_0_-mapping procedure would lead to accurate distortion estimates near moving targets and leads. Second, the added benefit of ECG over pulse oximetry remained uncertain when using online MRgRT-specific sequences. Phantom and volunteer measurements, treated in 2.3, 2.4 respectively, served to close these information gaps.

### 2.2 Patient population

At Radboudumc, UMCU, and MSKCC, online MRgRT workflows for CIED patients were clinically implemented in 2021–2023. Data was collected for 4 patients at Radboudumc (July 2023 – January 2024), 14 patients at UMCU (January 2022 – August 2023), and 3 patients at MSKCC (February 2023 – April 2024), each of whom had a CIED and received standard clinical care on a 1.5 T Unity MR-linac (Elekta AB, Stockholm, Sweden). At Radboudumc, informed consent was given retrospectively (protocol 2023–16850). At UMCU, data was retrospectively aggregated using internal approval by the institutional review board (‘FAST-ART’ protocol 20–519). At MSKCC, patients were analyzed under a retrospective IRB (protocol 21–129). For each patient, CIED risk class was determined, for both RT [3] and MRI [4]. Characteristics of the study population are listed in Table 1. Adverse events were collected based on reports of the safety checks that were performed by the CIED technologists immediately after each treatment fraction.

**Table 1:** Study population characteristics. Two patients were classified as MRI risk class IIa because the CIED/leads combinations were not tested for MRI conditionality, even though individually the CIEDs and leads were labelled MRI conditional in other combinations. For nine patients, radiotherapy risk was medium because of pacing dependence.

**Baseline characteristics**	
**Patients, n (%)**	**21 (100 %)**
Radboudumc	4 (19 %)
UMCU	14 (67 %)
MSKCC	3 (14 %)

**Age**^a^**, median (range)**	**71 (58–89)**

**Sex, n (%)**	
Male	19 (90 %)
Female	2 (10 %)

**Disease site, n (%)**	
Adrenal gland	2 (10 %)
5x8Gy	1 (5 %)
5x4Gy	1 (5 %)
Prostate	12 (57 %)
5x7.25Gy	11 (52 %)
5x8Gy	1 (5 %)
Pancreas	5 (24 %)
5x10Gy	1 (5 %)
5x7Gy	1 (5 %)
5x8Gy	3 (14 %)
Liver	1 (5 %)
3x15Gy	1 (5 %)
Oligometastases	1 (5 %)
3x10Gy	1 (5 %)

**CIED information**	

**Type, n (%)**	
Pacemaker	12 (57 %)
ICD	6 (29 %)
Pacemaker/ICD^b^	3 (14 %)
Monitor	0 (0 %)

**Pacing dependence, n (%)**	
Dependent	9 (43 %)
Independent	6 (29 %)
N/A^c^	6 (29 %)

**CIED risk classification**	

**Radiotherapy**[3]**, n (%)**	
Low risk	12 (57 %)
Medium risk	9 (43 %)
High risk	0 (0 %)

**MRI**[4]**, n (%)**	
Class I	19 (90 %)
Class IIa	2 (10 %)
Class IIb	0 (0 %)

a In years at start of treatment.

b CIEDs with both pacing and ICD functionality.

c N/A for CIEDs without pacing functionality (ICD and monitor).

### 2.3 Phantom measurements B_0_-mapping

To investigate the accuracy of B_0_-mapping for a lung tumor in respiratory motion near a CIED, a water-filled spherical target in the middle of a hollow acrylic insert was used (Supplementary Figure S1). In the first setup there was no CIED in the bore after which an unconnected MR-conditional ICD (Claria MRI, CRT-D SureScan, Model DTMA2D1, Medtronic Inc., MN, USA) was placed 175 mm, 105 mm, and 85 mm from isocenter, respectively. B_0_-maps were acquired with the (*static*) target at 5 positions (up to 10 mm from isocenter) and while simulating free-breathing (*dynamic*), employing a cos^4^ motion pattern at 15 breaths-per-minute and a 20 mm peak-to-peak amplitude [24].

Similarly, cardiorespiratory motion near a lead was simulated with a lead wrapped around a hollow cylindrical insert in three separate setups (leads details in Supplementary Table S1). Cardiorespiratory motion was simulated by adding a cardiac cos^4^ motion pattern. Static images were acquired at 7 positions.

Each of these phantom measurements was performed with a Quasar MRI^4D^ phantom (IBA Quasar, London ON) positioned at isocenter of a 1.5 T MR-linac. Phase-wraps were removed in each B_0_-map using a region-growing algorithm, utilizing both magnitude and phase maps [25]. Differences between static and dynamic B_0_-maps were compared voxel-wise.

**Fig. 1:**
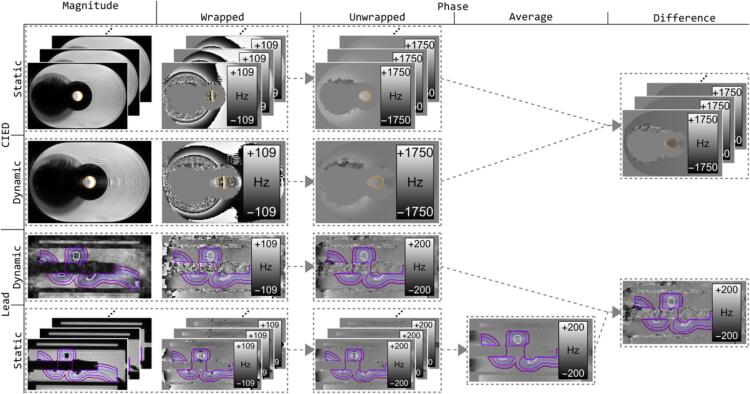
All acquired B0-maps contained phase-wraps that were unwrapped. For the CIED measurements, difference maps were calculated between the dynamic and each individual static B0-map. For the lead measurements, the static images were averaged prior to calculating a difference map with the dynamic image. For the CIED, images contain orange contours of the central sphere filled with liquid, simulating a lung tumor. For the leads, four purple contours are shown around the signal voids of the lead wrapped around an empty central cylinder. These contours were used for further analysis. (For interpretation of the references to colour in this figure legend, the reader is referred to the web version of this article.)

For the CIED measurements, this was done for voxels inside contours of the target at each static position, per configuration. For the leads, B_0_-maps were first averaged over static positions (see data processing pipeline in Fig. 1) and contours with increasing distance to the signal void around the lead were created (Supplementary Figure S2). Voxels inside signal voids were excluded.

Bland-Altman plots were created and mean and standard deviation calculated per CIED configuration. Mean and standard deviations were calculated for each distance to the lead-induced signal void. Using a typical read-out bandwidth of 450 Hz/mm, B_0_-values were translated into geometric distortions [19].

### 2.4 Volunteer ECG measurements

A healthy volunteer was connected to a patient monitor (Expression MR400, Philips, Best, The Netherlands) following clinical protocol and scanned on a 1.5 T MR-linac. Scans were acquired with three anatomical positions at isocenter: pelvis, abdomen, and thorax. MR planning sequences, including 3D T2 turbo spin-echo (TSE) and a 3D golden angle radial stack of stars (3D VANE) as well as diffusion-weighted imaging using an echo-planar sequence (DWI-EPI), part of routine MR-linac treatment, were acquired (Supplementary Table S2). 2D balanced fast field echo cine beam-on monitoring sequences were acquired along a one, two, or three orthogonal planes (conform local clinical practice). The ECG readout was qualitatively investigated after digitization and compared to the heart rate as automatically determined by the monitoring equipment.

## 3 Results

### 3.1 Clinical workflow

Our proposed clinical workflow consists of pre-treatment steps and online steps that are repeated during each fraction. In both cases, risk mitigation strategies from the MRI and RT guidelines were compatible and could readily be adopted, with MRI guidelines being the most stringent.

Based on the risk-assessment, the following adaptations were made for MRgRT in both the pre-treatment preparation and online workflow.

1. Each institute strictly monitored patient inclusion (e.g., patients were only eligible if there was a clear expected benefit) to justify the increased costs of online MRgRT.
2. Lacking the option to automatically alter sequences to adhere to CIED vendor MRI restrictions, scan protocols were manually adopted if necessary.
3. Given the similarities between treatment fractions, in terms of interaction between MR-linac and CIED, MRI compatibility verification and scan protocol adaptation was done only once per treatment course.
4. Thoracic disease sites were an absolute contra-indication pending clinical implementation of a method, like the one presented in Section 2.3, to estimate the clinical impact of geometric uncertainty of MRI nearby CIED or leads.
5. Each institute used pulse oximetry for cardiac monitoring and RT technologists (RTTs) were instructed to frequently contact the patient via the audio system. The in-room camera system (Radboudumc and UMCU) or a dedicated camera (MSKCC) was used to display the patient status inside the control room [11]. At MSKCC, ECG was also deployed and at UMCU an in-bore mirror system was used to monitor eye movement.
6. Use of pulse oximetry for patient monitoring was considered essential as ECG is prone to distortions when MR sequences are being acquired.

Division of tasks regarding CIED interrogation [3], [5] differed between institutions, depending on local practices. At Radboudumc, to reduce the workload, a CIED technologist was only present during the first and last treatment. During other fractions, the CIED interrogation was performed at the cardiology department and a registered nurse accompanied the patient to and from the RT department while monitoring the patient. At the UMCU, the cardiology and RT departments are nearby and so the CIED technologists performed the CIED interrogation at the MR-linac while cardiac monitoring was done by the RTT that performed the MRI. At MSKCC, a representative from the device manufacturer was present for CIED interrogation during each treatment fraction.

Using the clinical experience of the first treatments, some alterations in the clinical workflow with respect to medical supervision were made. At Radboudumc and UMCU it was initially decided to always have a radiation oncologist present during treatment in case a decision on pausing or breaking off the treatment was necessary due to a cardiac event. After initial experiences, it was decided that being on-call was sufficient as present personnel were trained and competent to both detect a cardiac event and perform an emergency procedure. At MSKCC, an advanced practice provider and/or the treating physician stayed for the duration of the treatment to monitor the patient from the control room.

### 3.2 Clinical experience

Twenty-one patients were treated for a total of 100 fractions with online MRgRT (see Table 1). One patient did not receive the last treatment fraction due to non-cardiac related complications (infection). One patient turned out to have had a minor cardiac event in between treatment days. Since this occurred frequently for this patient it was deemed to be unrelated to MRgRT. There were no records of adverse events during online MRgRT or abnormalities in CIED readings after each treatment fraction.

For all patients, estimated dose to the CIED was less than 2 Gy. For the 9 pacing dependent patients, RT-related risk was classified as intermediate with the rest classified as low risk. Similarly, MRI-related risk was low (Class I) for all but the two patients who had a CIED/lead combination that was not MRI-compatible (i.e., not tested) and as such were classified as Class IIa.

All patients had clear expected benefit of online MRgRT over conventional RT. Direct tumor visibility enabled dose escalation and/or reduced planning target volume margins. For one patient specifically, hypo-fractionated treatment reduced interruption of the use of methotrexate required for sarcoidosis.

### 3.3 Phantom measurements B_0_-mapping

**Fig. 2:**
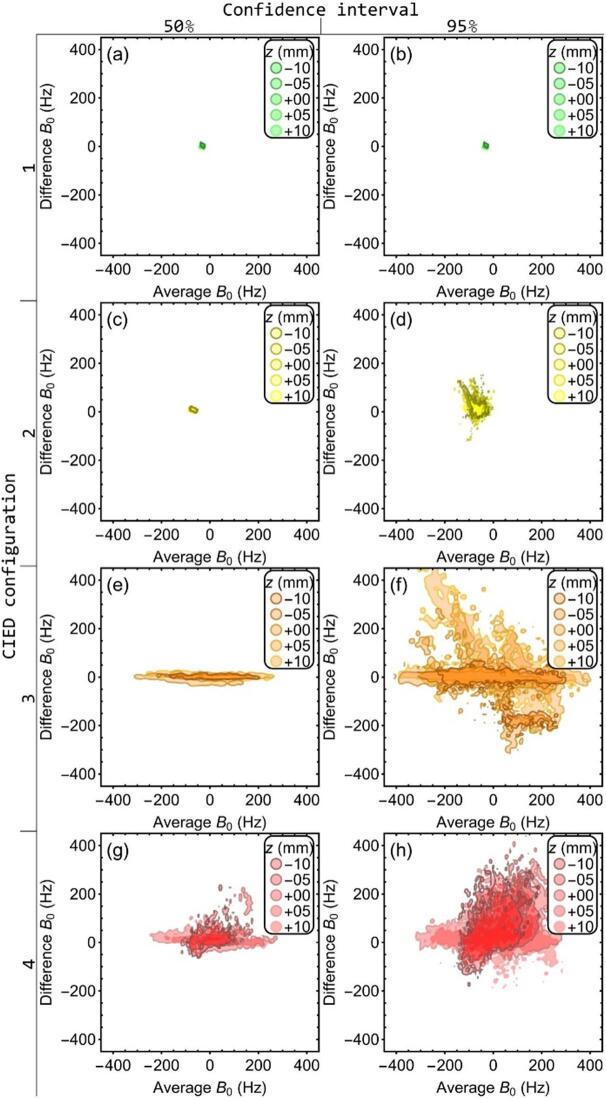
Bland-Altman plots showing 50 % (left) and 95 % (right) of the data for the four CIED configurations (no CIED in setup 1 and a CIED 175, 105, and 85 mm from isocenter, respectively, for setup 2 to 4). Each plot contains data comparing B0-maps acquired for the static case (sphere of interest at −10, −5, 0, 5, and 10 mm from isocenter) to the dynamic (moving phantom) case with a 20 mm peak-to-peak amplitude.

Differences in B_0_-values between static and dynamic measurements were compared using Bland-Altman plots (Fig. 2) per static position for each CIED configuration. For better visualization, data within the smallest regions containing 50 % (left) and 95 % (right) were shown, respectively. Introduction of the CIED increased the spread in both the average and difference B_0_-values. The CIED in configuration 3 and 4, closest to the target, distorted the B_0_-field severely enough that multiple phase-wraps occurred within the contoured region (Supplementary Figure S1). The observed average B_0_-offset ranged from −138 Hz to −52 Hz in setup 1 to −413 Hz to 411 Hz in setup 3. Groupwise mean differences between all static and dynamic measurements for the four CIED configurations were 4, 9, 1, and 48 Hz, respectively. The spreads, characterized by 1.96 times the standard deviation, were 39, 42, 149, and 161 Hz, equal to 0.09, 0.09, 0.33, and 0.36 mm, respectively.

**Fig. 3:**
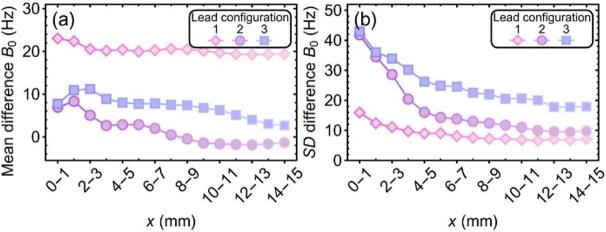
Mean difference (a) and the standard deviation of the difference (b) between the averaged B0-map for the static case and the dynamic (moving phantom) case for a range of distances from the signal void created by the lead.

The leads distorted the B_0_-field to a much lesser extent and more localized compared to the CIED. Still, some phase-wrapping occurred at some locations near the leads, most noticeable near the lead tip. Bland-Altman plots for the smallest (0–1 mm) and largest (14–15 mm) shells are shown in Supplementary Figure S3. Mean differences and standard deviations for all distances from the signal void are shown in Fig. 3. Maximum values for mean differences in this range for the three lead configurations were 23, 8, and 11 Hz, respectively. Maximum spreads, all at 0–1 mm from the signal void, were 16, 32, and 43 Hz, equal to 0.03, 0.07, and 0.10 mm, respectively.

### 3.4 Volunteer ECG measurements

At rest, the volunteer had a steady heart rhythm around 70 beats per minute (bpm). Four-second ECG readouts, together with automatically determined ECG-based heart rates are shown in Supplementary Figures S4 and S5.

Heart rate was between 60 bpm and 80 bpm at the pelvic location for the 3D T2 and DWI, at the abdominal position for the 3D T2 and coronal 2D cine, and at the thoracic position for the coronal 2D cine, DWI, and for both the balanced and unbalanced T1w 3D VANE. The automatically determined heartrate was 193 bpm at the pelvic position for the two-plane 2D cine and 177 bpm for the DWI at the abdominal position. For two acquisitions, the sagittal 2D cine at the pelvic position and the two-plane 2D cine at the thoracic position, a heart rate could not be determined.

All ECG-readouts acquired during scanning contained severe signal distortions. Manual detection of the graphical visual deflections in the ECG signal (QRS complex) was possible by an inexperienced reader (R.K.) for the baseline acquisition (on table outside bore) and for the 3D T2 planning sequence at the pelvic and abdominal positions, however.

## 4 Discussion

The B_0_-mapping procedure, based on previous work [20], [21], was extended for thoracic use based on results from the 4D phantom measurements. From these measurements, we concluded that B_0_-maps acquired during free-breathing are sufficiently accurate in the region without phase-wraps. If phase-wraps are present in the target area then, considering the larger uncertainties in the B_0_-maps, the magnitude of the distortion likely results in clinically relevant geometrical shifts. Conservatively, we would therefore propose a qualitative approach where eligibility for MRgRT is based on the presence of phase-wraps in the target area in B_0_-maps acquired during pre-treatment MRI on a per patient basis.

Apart from geometric distortions in the target area, geometric distortions in surrounding tissue might also affect the treatment quality. Image deformation can result in altered dose-volume histogram statistics for organs at risk, used to optimize and evaluate treatment plans. Additionally, there might be a dosimetric effect, for example when the external contour is deformed (thereby changing radiological depth). However, these effects are expected to be secondary to deformations and shifts of the target itself.

Volunteer measurement confirmed large errors can occur in ECG-readout inside an MRI environment, depending on body positioning and sequences being acquired [5]. Pacemakers are typically programmed in an asynchronous mode with maximal output possibly increasing the cardiac-related signal in the ECG-readout compared to that of a healthy volunteer. For patients with a CIED without pacing capability, however, similar levels of distortions are expected and therefore it is advised to verify ECG functionality during pre-treatment MRI on a per patient basis.

ECG signal distortions depend on timings of gradient-fields and radio-frequency pulses which vary during the acquisition of an MRI sequence, particularly for DWI. This effect was not investigated in this study. Furthermore, only one CIED was used for phantom measurements. Since the pathway of creating distortions is independent of the CIED type, however, we argued it would suffice to validate our method.

In conclusion, CIED patients received MRgRT on a 1.5 T MR-linac and a similar workflow can be readily implemented at other institutes by following existing MRI and RT guidelines. Treatment eligibility of thoracic patients with a CIED can be determined using a B_0_-mapping procedures to assess geometric fidelity. ECG-based cardiac monitoring might be heavily distorted during MRI acquisition, depending on positioning of the patient in the bore and the sequences used. Therefore, pulse oximetry is indispensable if cardiac monitoring is needed.

## Declaration of Generative AI and AI-assisted technologies in the writing process

During the preparation of this work the authors used ChatGPT and Copilot to improve readability and language. After using this tool/service, the authors reviewed and edited the content as needed and take full responsibility for the content of the publication.

## Declaration of competing interest

The authors declare that they have no known competing financial interests or personal relationships that could have appeared to influence the work reported in this paper.
