# Supplementary material for: Multi-institutional experience treating patients with cardiac devices on a 1.5 Tesla magnetic resonance-linear accelerator and workflow development for thoracic treatments
Source: Phys Imaging Radiat Oncol. 2024 Nov 26;32:100680. doi: 10.1016/j.phro.2024.100680 (PMC11636337; doi:10.1016/j.phro.2024.100680)
Supplement: Supplementary Data 1 [file mmc1.docx]

# Appendices

## Supplementary tables

Supplementary Table S1: Basic properties of the leads used in the 4D phantom measurements, where lead 1-3 were used for configurations 1-3 (see Fig 5. in the appendix), respectively.

|  | **Length, cm** | **Diameter, mm** | **Type** | **Vendor** | **Model** |
| --- | --- | --- | --- | --- | --- |
| **Lead 1** | 58 | 2.0 | Pacing | Medtronic | 5076 |
| **Lead 2** | 88 | 1.3 | Pacing | Medtronic | 4796 |
| **Lead 3** | 55 | 2.8 | Defibrillation | Medtronic | 6947M |

Supplementary Table S2: Details on MRI sequences (MR software version 5.7) acquired during ECG read-out.

|  | **Total**  **scan time (min:sec)** | **Acquisition voxel size  (mm^3^)** | **FOV   (mm^3^)** | **TR/TE   (ms)** | **Flip angle  (°)** | **Read-out bandwidth^a^  (Hz/mm)** | **b-values  (s/mm^2^)** |
| --- | --- | --- | --- | --- | --- | --- | --- |
| **3D-T2** | 3:41 | 1.1x1.1x2.0 | 400x448x250 | 1300/87 | 90 | 694 |  |
| **2D cine** | 1:00 | 3.0x3.0x5.0 | 400x424x5 | 3.4/1.7 | 40 | 1076 |  |
| **3D VANE** | 3:00 | 1.8x1.8x3.0 | 500x500x220 | 3.3/1.3 | 40 | 944 |  |
| **DWI** | 3:00 | 3.5x3.5x4.0 | 420x420x100 | 3760/180 | 90 | 26 | 0, 150, 500 |

^a^For EPI sequences, field-related geometric distortions are in the phase-encoding (PE) direction. Therefore, bandwidth in the PE-direction was reported.

## Supplementary figures


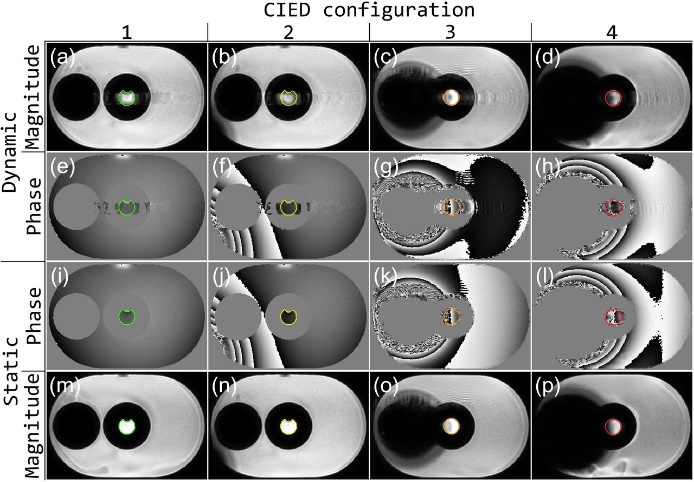


Supplementary Figure S1: Transversal images of the magnitude and phase images from the B_0_ mapping procedure are shown for the four CIED configurations for both the moving (dynamic) and static phantom. From left to right; no CIED (1), CIED at 175 mm from isocenter outside the phantom (2), CIED inside phantom at 105 mm from isocenter (3), and CIED inside phantom at 85 mm from isocenter. Phase wraps can also occur in the absence of a CIED near an air-water interface but are most noticeable in the region around the signal void. Movement of the phantom also introduces imaging artifacts, here in the left-right direction.


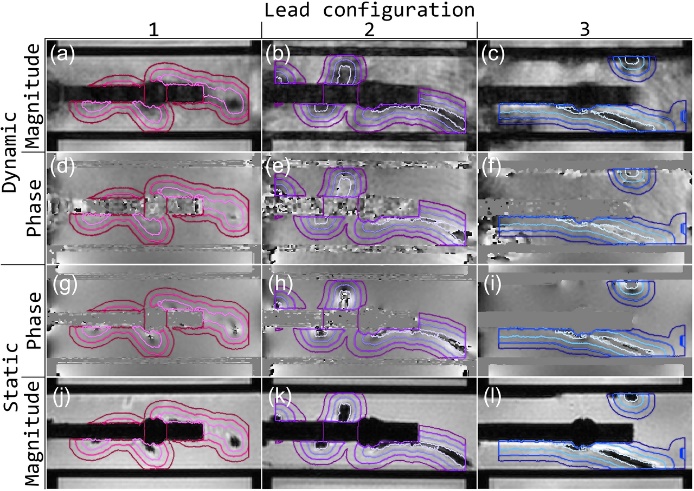


Supplementary Figure S2: Coronal images of the magnitude and phase images from the B_0_ mapping procedure are shown for the three lead configurations for both the moving (dynamic) and static phantom. Each lead has been coiled differently around the central cylinder to capture various configurations. Movement of both the lead and the phantom itself create a blurring effect in the B_0_ map.


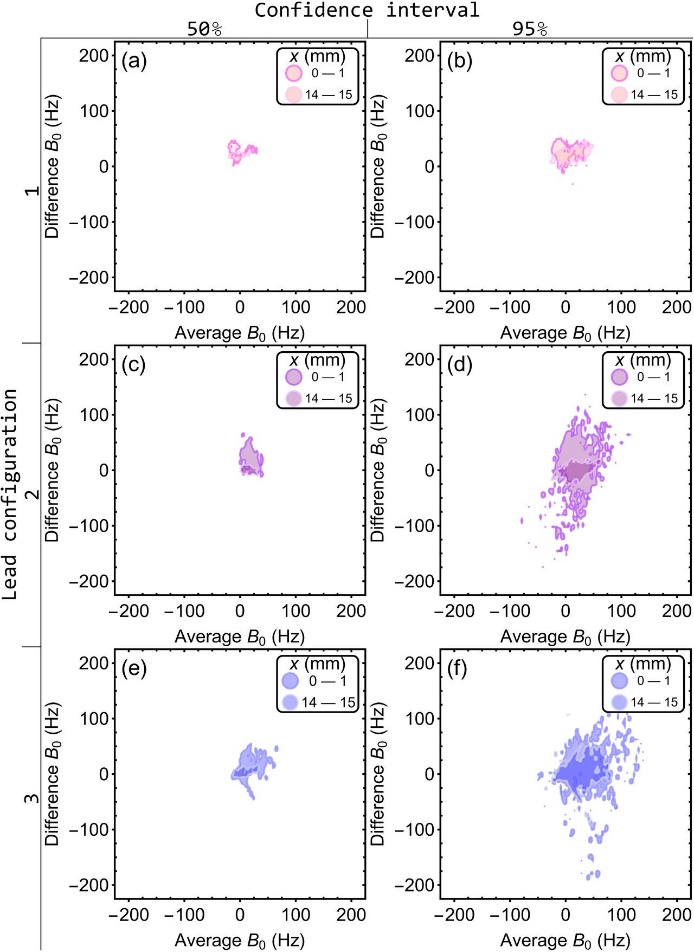


Supplementary Figure S3: Bland-Altman plots showing 50% (left) and 95% (right) of the data for the three lead configurations. Each plot contains data comparing an averaged B_0_ map for the static case to the dynamic (moving phantom) case, at a distance 0-1 mm and 14-15 mm from the signal void created by the lead.


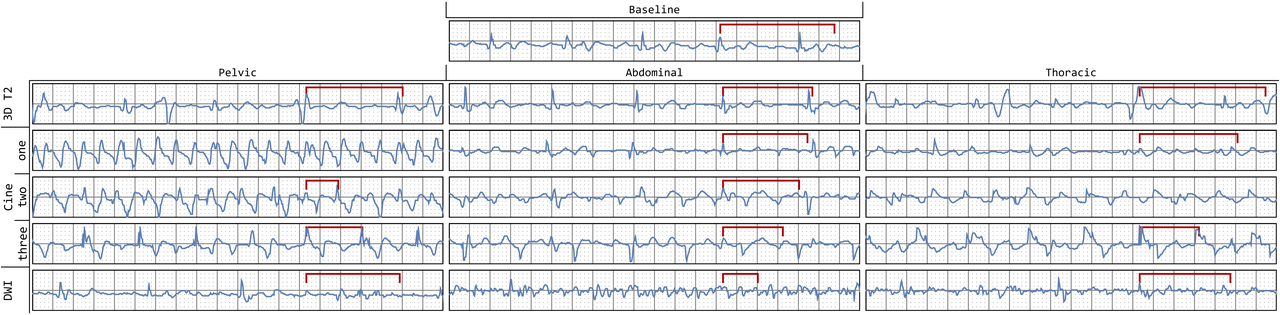


Supplementary Figure S4: Four-second ECG read-out samples at baseline and at a pelvic, abdominal, and thoracic scanning position during acquisition of 3D T2, Cine (one-, two-, and three-planes), and DWI MRI’s. Average time between pulses (heart rate), as automatically determined by the ECG equipment, is shown in red when applicable.


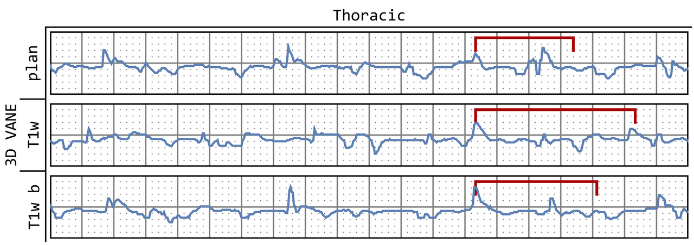


Supplementary Figure S5: Four-second ECG read-out samples for three types of 3D VANE MRI acquisitions at the thoracic scanning position. Average time between pulses (heart rate), as automatically determined by the ECG equipment, is shown in red.
